# Effects of Yacon on Colonic IFN-γ and Goblet Cells of the 2,4,6-Trinitrobenzene Sulfonic Acid-Induced Colitis Mouse Model

**DOI:** 10.29252/ibj.24.5.276

**Published:** 2020-01-20

**Authors:** Larasti Putri Umizah, Widya Wasityastuti, Dewiyani Indah Widasari, Setyo Purwono

**Affiliations:** 1 Master in Biomedical Sciences, Faculty of Medicine, Public Health, and Nursing, Universitas Gadjah Mada, Indonesia;; 2 Department of Physiology, Faculty of Medicine, Public Health and Nursing, Universitas Gadjah Mada, Indonesia;; 3 Department of Anatomical Pathology, Faculty of Medicine, Public Health, and Nursing, Universitas Gadjah Mada, Indonesia;; 4 Department of Pharmacology and Therapy, Faculty of Medicine, Public Health, and Nursing, Universitas Gadjah Mada, Indonesia

**Keywords:** Anti-Inflammatory, Colitis, Goblet cells, IFN-γ, Supplementation

## Abstract

**Background:**

IBD is a chronic inflammatory condition associated with damage to the intestinal mucosal barrier. Supplementation of yacon tubers has been known to have positive effect on intestinal health. Therefore, we conducted the present study to investigate the effect of yacon tuber powder on Th1 activation pathway by evaluating IFN-γ levels and the number of goblet cells in the colon of colitis mouse models.

**Methods:**

Thirty BALB/c mice were divided into five groups: 1, control group (G1) and 2-5, TNBS-induced colitis groups (GII-GV). Yacon powder was given to three of the TNBS-induced colitis groups with the doses of 0.165 g/30 g BW (GIII), 0.331 g/30 g BW (GIV), and 0.662 g/30 g BW (GV) for 14 days. IFN-γ levels were assessed using ELISA, while number of goblet cells was calculated based on histological observation.

**Results:**

Significantly lower IFN-γ levels was observed in GV compared to colitis group (GII) (*p* = 0.007). GV also showed significantly higher number of goblet cells per 100 epithelial cells in 20 crypts (*p* = 0.000) than GII.

**Conclusion:**

The administration of yacon powder at a dose of 0.662 g/30 g BW could decrease IFN-γ levels and improve the healing of intestinal mucosa in colitis mouse models by increasing the number of goblet cells.

## INTRODUCTION

Inflammatory bowel disease is recognized as a chronic inflammation of the gastrointestinal tract with two main clinical subtypes, ulcerative colitis and Crohn's disease. The exact cause is still unknown, but it is known that IBD is associated with damage to the intestinal mucosal barrier. The resulting adhesion and invasion of luminal microorganisms trigger immune response in the intestinal tissue^[^^1 ▶^^,^^2 ▶^^]^. In normal condition, intestinal immune system activates T regulator cells, which stimulates the production of anti-inflammatory IL-10 and TGF-β. However, in IBD patients, the innate immune system recognizes mucosal colonic cells as antigens^[^^2 ▶^^]^. Dendritic cells will present luminal and intra-mucosal antigens to immune effector cells and produce IL-12, which promotes the differentiation of naïve CD4^+ ^T cells into Th1. The activated Th1 cells produce IFN-γ, resulting in the activation of macrophages. These macrophages could damage intestinal epithelium, leading to the reduction in the number of goblet cells. The production of IFN-γ by Th1 can also lower the number of goblet cells by inhibiting the maturation of these cells^[^^3 ▶^^]^.

Intrarectal TNBS application in low doses together with ethanol can induce colitis in BALB/c strain mice^[^^4 ▶^^]^. The ethanol disrupts the intestinal epithelial barrier, while TNBS acts as a hapten that can enter the lamina propria and bind to tissues, rendering normal tissue immunogenic to the host immune system. This “haptenization” of colonic proteins triggers inflammation by inducing Th1 responses and high IFN-γ production^[^^4 ▶^^,^^5 ▶^^]^. IFN-γ activates macrophages and produces NO, which can interact with O2^-^ anions and form ONOO^-^ ions. The cytotoxic nature of ONOO^-^ can cause damage to healthy intestinal tissue, including intestinal crypts along with other intestinal components, resulting in a decreased number of goblet cells and increased inflammatory response^[^^1 ▶^^,^^6 ▶^^]^.

Nowadays, medicinal plants or their active components have become alternatives to reduce inflammation, heal and protect the intestinal mucosa in IBD patients who are not responsive to steroid drugs or are not willing to take standard drugs. Their limited toxicity further promotes the use of medicinal plants as therapy for IBD^[^^7 ▶^^]^. 

Yacon is one of the medicinal plants that contains abundant FOS in its tubers^[^^8 ▶^^]^. FOS is a prebiotic suggested to improve intestinal health, but studies on the prebiotic effects of FOS from yacon tubers is rare^[^^9 ▶^^]^. A previous research on healthy mice reported that daily consumption of FOS from yacon does not have a negative impact on the body's immune system^[^^10 ▶^^]^. FOS even has anti-inflammatory effects, improves the mucosal immune system and decreases the risks associated with autoimmunity and metabolic diseases. However, it is not yet known whether powdered yacon tubers can affect the production of the IFN-γ cytokines and the number of goblet cells in the colon tissue of a TNBS-induced colitis model in BALB/c strain mice. This study investigated the prebiotic influence of yacon powder on IFN-γ levels and the number of goblet cells in colon tissue of TNBS-induced mice. We hypothesized that the administration of yacon powder would lower IFN-γ levels and increase the number of goblet cells in the TNBS-induced mouse model. 

## MATERIALS AND METHODS

**Animal model**

Thirty male BALB/c mice, aged 6-8 weeks and weighing 28-32 g, were obtained from LPPT and were used as experimental animals. The mice were placed in sterile cages with lids. The cage floor was covered in husks. The cages were equipped with food and drink containments. Light cycle of 12 hours of light and 12 hours of darkness was maintained with the room temperature of 24-26 ^o^C and humidity of 60-65%. Mice were fed *ad libitum*, which was controlled every day, and were given seven days to adapt before the treatment was started. The animals were divided into five treatment groups, namely group I (GI), II (GII), III (GIII), IV (GIV), and V (GV). GI was the control group of healthy mice, group II included the case control group (induced colitis mouse model), and GIII, GIV, and GV were a group of colitis mouse model that were given yacon powder at various doses of 0.165, 0.331, and 0.662 g/30 g BW, respectively. 

**Colitis induction using TNBS**

The induction of colitis in GII, GIII, GIV, and GV mice was performed by intrarectal application of TNBS (Sigma-Aldrich®, USA), based on the method described by Steinhoff and Visekruna^[^^4 ▶^^]^. To make intrarectal solution, TNBS was dissolved in 70% ethanol (2.5% w/v), and then a total volume of 150 μL was administered using a lubricated (Durex KY Jelly®, Indonesia) 4-cm probe. After TNBS administration, mice were left in a vertical position for 60 seconds to avoid TNBS reflux. On the 7^th^ day after induction, mice in GII group showed symptoms of colitis, characterized by diarrhea and weight loss, as well as apparent intestinal tissue damage from tissue histology analysis.

**Yacon diet supplement**

Dietary supplements of yacon powder (Yacon Pure *Smallanthus sonchifolius*®, Only Natural, USA) were given to GIII, GIV, and GV as an intervention for colitis. The doses of yacon powder were obtained from a previous study conducted by Genta *et al.*^[^^11 ▶^^] ^in healthy mice. The yacon powder was dissolved in water and then given to the mice by oral gavage with a volume of 0.33 ml daily for 14 days.

**Tissue collection**

Collection of the colon tissue was conducted after the 14-day treatment. The mice were anesthetized using ketamine (Ivanes ketamine HCl injection 100 mg/10 ml®, Indonesia) and then euthanized by cervical dislocation. Colon tissue was then taken for further analysis. The tissues were divided into two portions: one was immediately placed in containers containing 4% PBS-formalin for histological examination, while the other portion was washed with PBS (pH 7.4) and was directly processed according to the sample preparation protocol for ELISA examination using an ELISA kit (Fine-test®, USA).

**Histological staining**

The obtained colon tissues were made into paraffin blocks through several stages, dehydrating, clearing, and embedding. The paraffin blocks were then sectioned to a thickness of 3 μm and affixed to an object-glass. The slides were stained with PAS staining, which was made according to the protocol of Lynch *et al.*^[^^12 ▶^^]^.

**Fig. 1:**
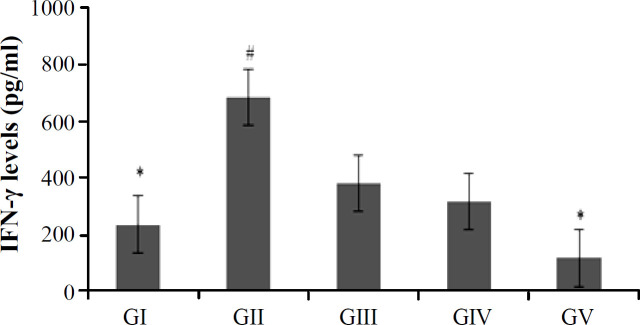
IFN-γ levels in colonic tissue of mice. Data are presented as mean ± SD (n = 5). GI (control), GII (TNBS), GIII (TNBS + yacon 0.165 g/30 g BW), GIV (TNBS + yacon 0.331 g/30 g BW), GV (TNBS + yacon 0.662 g/30 g BW). ^#^ and ^*^ show significantly different from GI and GII, respectively  (*p* < 0.05).

**Histology analysis**

Six PAS-stained longitudinal histological sections of colon from each group were observed using a light microscope (Olympus CX21®, Phillipines) and randomly photographed until a total of 20 crypts were obtained from each section. From the photographs, the total number of PAS-positive cells was counted per 100 epithelial cells along with those 20 longitudinally sectioned crypts. The average number of goblet cells per 100 epithelial cells from each section was then calculated based on the method provided by Bergstrom *et al.*^[^^13 ▶^^]^.

**Biochemical examination**

 IFN-γ levels were tested using the ELISA method. The procedure was carried out according to the protocol in the ELISA kit. The procedure was started with preparation of standard solutions (dilution), samples (dilution), and control. Next, the plates were washed with a wash buffer, followed by the adding the standard solutions, samples, and controls into the corresponding wells. The secondary antibodies, substrates, conjugates, and stop solutions were then added in turn. The OD/absorbent was read at 450 nm on a microplate reader, immediately after adding the stop solution. 

**Statistical analysis**

 The data obtained were analyzed using SPSS software (IBM Corp., Chicago). Results were expressed in mean ± SD. Results of the different treatment groups were analyzed using ANOVA variant analysis, continued with the post-hoc test. The degree of statistical significance was set at *p* < 0.05, and the confidence interval was 95%.

**Ethical statement**

The above-mentioned sampling/treatment protocols were approved by the Ethics Committee for Animal Experiment of LPPT, Universitas Gadjah Mada, Indonesia (reference No. 00003/04/LPPT/IV/2019).

## RESULTS

**IFN-γ levels**

The statistical analysis of IFN-γ levels (Fig. 1) indicated that there were significant differences between GI and GII (*p* = 0.049). However, GI was not significantly different from the three yacon-treated groups. GV was significantly lower than GII (*p* = 0.007), but there were no significant differences between GII, GIII, and GIV.

**Goblet cell counts**

**Fig. 2.**
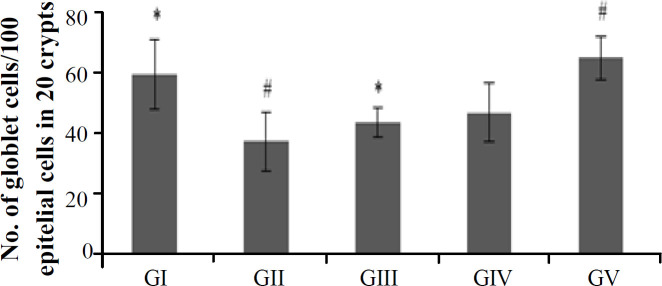
The number of goblet cells per 100 epithelial cells. Data are presented as mean ± SD (n = 5). GI (control), GII (TNBS), GIII (TNBS + yacon 0.165 g/30 g BW), GIV (TNBS + yacon 0.331 g/30 g BW), GV (TNBS + yacon 0.662 g/30 g BW). ^#^ and ^*^ show significantly different from GI and GII, respectively (*p* < 0.05)

**Fig. 3.**
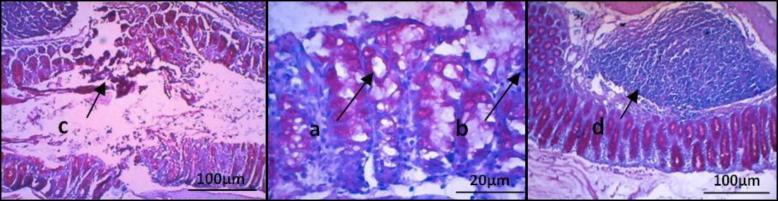
Histology of colonic tissue in the TNBS-induced colitis model in mice (GII). (a) goblet cell necrosis (scale 20 μm), marked by empty spaces in the crypts; (b) leukocyte infiltration (scale 20 μm) marked by leukocytes in the inflamed area; (c) transmural inflammation (scale 100 μm) marked by inflammation throughout all layers of the colon; (d) active lymphoid follicles (scale 100 μm), marked by its large and solid shape

The results of goblet cells count per 100 epithelial cells in colon tissue with PAS staining are presented in Figure 2. The statistical analysis results showed a significant difference between GI and GII (*p* = 0.001). By comparison GI with GIII, we observed a significant difference (*p* = 0.024), but the results in GIV and GV were not significantly different from GI. When comparing GII to GV, significant difference was also observed (*p* = 0.000). In the treatment groups given yacon powder with three different doses, there were statistically significant differences between GV and GIV (*p* = 0.011) and GIII (*p* = 0.003), but no significant differences were found between GIII and GIV. In the PAS-stained histological slides of colon tissue from TNBS-treated mice in Figure 3, there was necrosis of goblet cells, leukocyte infiltration, transmural inflammation, and the presence of active lymphoid follicles, indicating active inflammation in the colon of these mice. 

**Fig. 4:**
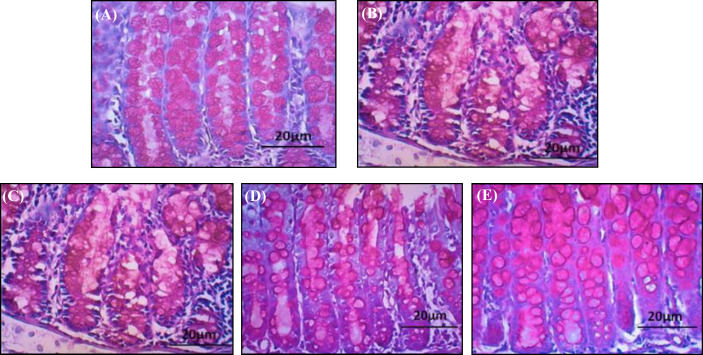
Histologic examination of PAS-stained colonic tissue. Colonic tissue (scale 20 μm) from (A) GI/control; (B) GII/TNBS;  (C) GIII/TNBS + yacon 0.165 g/30 g BW; (D) GIV/TNBS + yacon 0.33 1 g/30 g BW; (E) GV/TNBS + yacon 0.662 g/30 g BW

The results of histological analysis among the groups showed neither tissue nor crypt damage in GI group. The goblet cells had normal shape and color, reflecting healthy colon tissue (Fig. 4A). The same results were not seen in the GII group (Fig. 4B). In GII, the damage of colon tissue due to TNBS administration was shown by the necrosis of goblet cells, resulting in a decreased number of goblet cells. In GIII group, histological examination revealed leukocyte infiltration and necrosis of goblet cells, which was displayed by the presence of empty spaces in the crypts (Fig. 4C). Meanwhile, in the GIV treatment group, the damaged tissue had begun to improve. The improvement was observed in the shape and color of the goblet cells, but the number of goblet cells remained low and leukocyte infiltration was still apparent (Fig. 4D). In GV, the colon tissue relative to GIII had improved, which was demonstrated by the better shape and increased number of goblet cells, and the absence of active lymphoid follicles (Fig. 4E). This change represented no active inflammation in the colon tissue of GV. 

## DISCUSSION

According to the results, GII had a significantly higher IFN-γ level than GI, indicating that intrarectal administration of TNBS along with ethanol successfully induced colitis in mice. Ethanol damaged the intestinal epithelial barrier, which leads to the adhesion and invasion of microorganisms on the surface of the epithelium. TNBS could then couple with intestinal protein, causing them to become immunogenic to the host’s immune system and triggering Th1-mediated immune response^[^^4 ▶^^,^^5 ▶^^]^. Innate immune system will present intra-mucosal antigens and produce IL-12, which promotes the differentiation of naïve CD4^+ ^T cells into Th1. The production of IFN-γ by Th1 can lower the number of goblet cells by inhibiting the maturation of the cells^[^^3 ▶^^]^. As IFN-γ is expressed in Th1 pathway, we examined its levels to see the effect of yacon administration on it.

The IFN-γ levels in the three yacon-treated groups did not differ significantly compared to the control group. These results indicate that the administration of yacon powder could provide anti-inflammatory effects. Yacon powder contains prebiotics such as FOS that cannot be degraded by the digestive system of humans and animals. However, the beneficial bacteria such as Bifidobacterium in the gut produce β-fructosidase enzyme, which can ferment FOS into SCFA. SCFA inhibits NF-kB in producing IFN-γ, resulting in the higher production of anti-inflammatory IL-10, which in turn can prevent inflammation^[^^14 ▶^^,^^15 ▶^^]^. Prebiotics also function as a supplement for the growth and development of beneficial microorganisms. An increase in the activity and the number of beneficial bacteria in the colon can suppress the growth of pathogenic bacteria, prevent the attachment of pathogens in the intestinal mucosa, improve intestinal barrier function by strengthening the tight junctions between intestinal epithelial cells, and maintain intestinal crypts and goblet cells^[^^16 ▶^^-^^18 ▶^^]^.

An elevation in beneficial bacteria in the intestine can also act as an immuno-modulator by triggering the differentiation of effector T cells into Treg and Th2, which both can then trigger the production of anti-inflammatory IL-10. Th2 cells enhance the production of TGF-β, causing the differentiation of B cells into plasma cells. Differentiated B cells will experience isotype switching to IgA to inhibit bacterial invasion^[^^19 ▶^^,^^20 ▶^^]^. Activation of Th2 is also known to suppress Th1 and inhibit IFN-γ production, maintaining the intestinal condition in a balanced state and preventing inflammation^[^^21 ▶^^]^.

Insignificant lower expressions of IFN-γ than the colitis-induced group were observed in GIII and GV. Lower IFN-γ levels in GIII, GIV, and GV may be due to the presence of acetate and butyrate in yacon. Acetate and butyrate are SCFA produced from prebiotic fermentation in the intestine. Acetate can induce apoptosis in colon cancer cells^[^^22 ▶^^]^, whereas butyrate can prevent inflammation by blocking the NFκB pathway, resulting in the suppression of IFN-γ production^[^^23 ▶^^]^.

GII had a significantly lower number of goblet cells in the colon tissue than GI, showing that colitis decreased the number of goblet cells. This reduction occurs because TNBS causes the misidentification of the intestinal mucosa as an immunogenic antigen. This condition initiates an immune response to attack the intestinal mucosal components, in which reduces the number of goblet cells^[^^4 ▶^^]^. Moreover, high production of IFN-γ by Th1 might correlate with the increased NO level produced by active macrophages^[^^24 ▶^^]^. High NO levels are dangerous and can damage healthy intestinal tissue owing to the interaction between NO and the O_2_^-^ anion, which is produced in colitis patients. Both will react and form ONOO^-^, which is more dangerous and cytotoxic than NO and O_2_^-[^^25 ▶^^]^. In a research conducted by Yue *et al.*^[^^26 ▶^^]^, it was also reported that an increase in NO and ONOO^-^ production contributes to the apoptosis of intestinal epithelial cells (including goblet cells) during inflammation, causing a decrease in the number of goblet cells. This explanation is supported by the histological analysis of the colon tissue from mice induced by intrarectal TNBS- ethanol. The results of an earlier study confirm that TNBS can induce colitis in mice, as characterized by goblet cell necrosis, leukocyte infiltration, active lymphoid follicles indicating active inflammation, and transmural inflammation, an inflammation that occurs in all layers of the intestine^[^^27 ▶^^]^. However, in GIV and GV, the average numbers of goblet cells were comparable to GI. This similarity shows that the administration of yacon powder could enhance the number of goblet cells in both groups, while the results in GIII were still significantly different from GI. Prebiotics are known to be able to maintain intestinal crypts and goblet cells^[^^18 ▶^^]^. When comparing IFN-γ levels and the average number of goblet cells, the IFN-γ levels in the three treatment groups were relatively close to the level of GI, but the average number of goblet cells in GIII still showed a decrease compared to GI. This finding may be caused by the fact that IFN-γ turnover occurs in 46 hours^[^^28 ▶^^]^, quicker than goblet cells, whose turnover takes 72 hours. The longer turnover time for goblet cells is due to the maturation process needed to become a mature goblet cell, which is shaped like a goblet and filled with mucin^[^^29 ▶^^]^. 

In conclusion, yacon powder administration at a dose of 0.662 g/30 g BW has positive effects on TNBS-induced mouse models by significantly lowering IFN-γ level and increasing the number of goblet cells.
